# Mooring observed intraseasonal oscillations in the central South China Sea during summer monsoon season

**DOI:** 10.1038/s41598-021-93219-3

**Published:** 2021-07-01

**Authors:** Sen Jan, Ming-Huei Chang, Yiing Jang Yang, Chung-Hsiung Sui, Yu-Hsin Cheng, Yu-Yu Yeh, Chung-Wei Lee

**Affiliations:** 1 Institute of Oceanography, National Taiwan University, Taipei, Taiwan, ROC; 2 Department of Atmospheric Sciences, National Taiwan University, Taipei, Taiwan, ROC

**Keywords:** Ocean sciences, Physical oceanography

## Abstract

The South China Sea (SCS) is a high biodiversity region in the world ocean, supports abundant marine resources to the peripheral nations, and affects weather/climate in southeast Asia. A better understanding of its circulation is important to better prediction and management of the SCS. Here we reveal sizable intraseasonal oscillations at period ~ 50 days between May and November 2017 in the acoustic Doppler current profiler observed velocity in the central SCS. Satellite observed wind and sea level data together with a process-oriented numerical experiment suggest that the oscillations were caused by locally-generated and remotely-penetrated westward-propagating Rossby waves. The summer southwesterly monsoon strengthening/weakening and the resultant Ekman pumping velocity and shoreward Ekman transport increase/decrease and consequent coastal sea level rise/fall off the west coast of Palawan create westward-propagating Rossby waves causing velocity oscillations in the central SCS. Besides the local generation, Rossby waves with sea level anomaly > 0.2 m propagating from the Pacific through the Sulu Sea into the SCS could contribute to the intraseasonal velocity oscillations in the central SCS.

## Introduction

**Figure 1:**
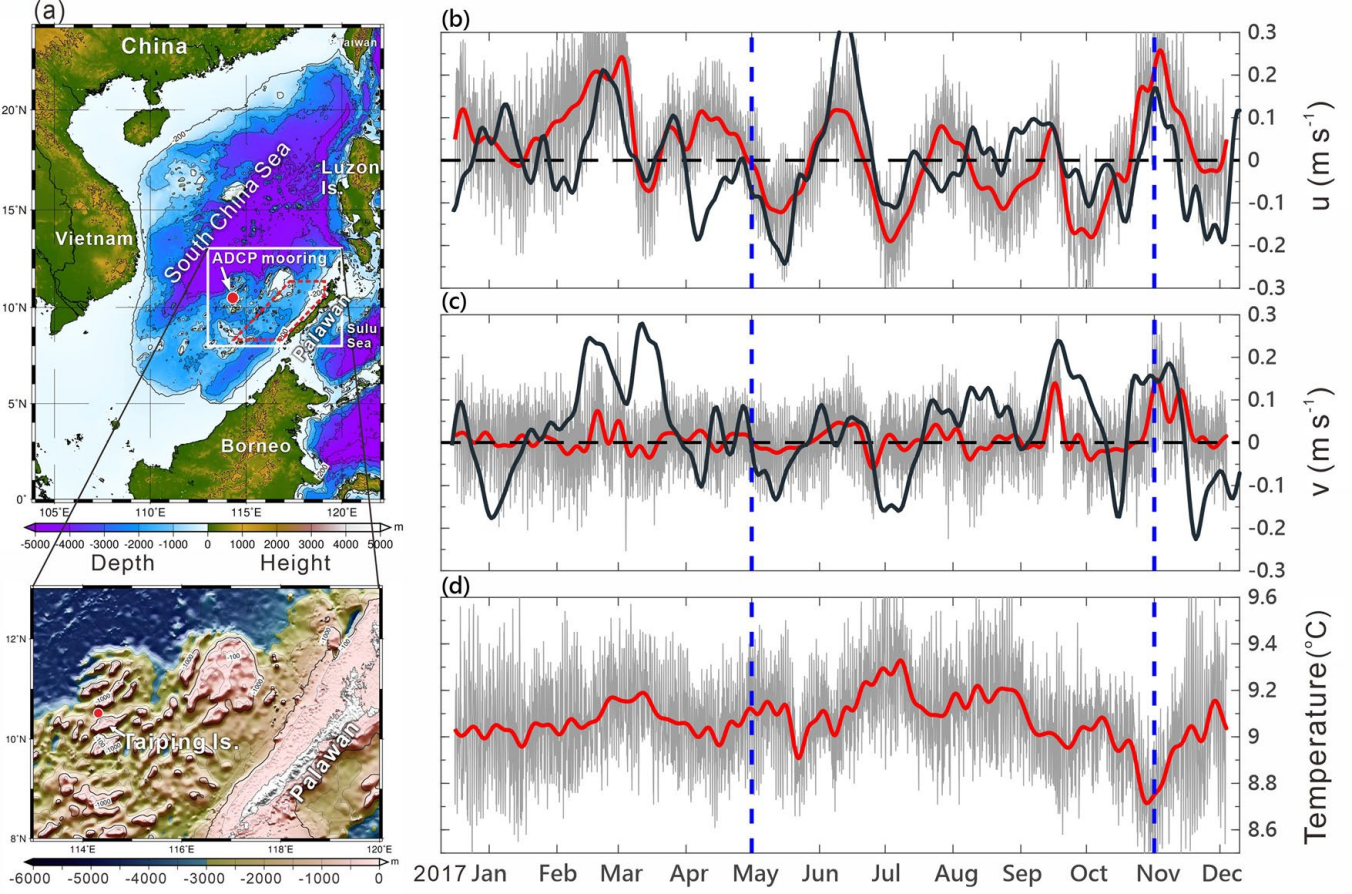
Bathymetry of the SCS and the moored ADCP observed velocity and temperature. (**a**) Bathymetric chart of the SCS and an enlarged bathymetry in the lower panel showing the topography around the mooring. The filled red circle in both panels indicates the mooring location. (**b,c**) Components of the depth-averaged (40–120 m) velocity (gray curves). The 8.33-day low-pass-filtered, satellite altimeter data derived geostrophic currents (black curves) are overlaid on (**b,c**). The polygon area enclosed by the red dashed lines in (**a**) is for the area average of satellite SLA, wind stress, and wind stress curl. (**d**) Raw temperature at 480 m (gray curve). The depth-averaged velocity and raw temperature data are 8.33-day low-pass filtered (red curves).

The South China Sea (SCS; Fig. 1a), an important maritime route between the Indian and Pacific Oceans, provides substantial marine resources to the peripheral nations. The circulation in the SCS and the air-sea interactions it involves are crucial to influence weather/climate in southeast Asia. Improving the understanding of the SCS circulation and the underlying physical processes are not only for sustainable management but also for better numerical prediction of the SCS. This study is thus aimed to advance our understanding of the SCS circulation and underlying dynamics.

The circulation in the SCS is subject to the variability of winds, which are controlled by the Asian monsoon, southwesterly in summer and northeasterly in winter, and are influenced by multiscale climate oscillations^1^. Within the broad timescales, the boreal summer intraseasonal oscillation (BSISO) is a pronounced atmospheric activity, which contains convection anomalies initiated in the central Indian Ocean that propagate eastward across the Maritime Continent and northward in the SCS^2^. As positive convection anomalies propagate northward from the Bay of Bengal in the Indian Ocean to the SCS, they cause westerly anomalies near and to the south and easterly anomalies to the north of the convective center^3,4^. The westerly anomalies enhance the southwest monsoon across the SCS during boreal summer.

Driven by the East Asian monsoon, the basin-wide SCS circulation is an anticyclonic gyre in summer with an offshore jet leaving the Vietnam coast at ~ 12° N and a cyclonic gyre in winter with a pronounced southward-flowing current along the Vietnam coast^5–9^. Two seasonally varied cyclonic eddies were further revealed in the circulation: One located off the Vietnam coast during late summer through early fall and the other off the northwest coast of Luzon Island during late fall through early spring^10^. The formation of the two seasonal mesoscale eddies is attributed to the basin-wide circulation and the wind stress curl-induced Ekman pumping^10^.

At intraseasonal timescale, a 45-day variation in the summertime offshore jet off Vietnam was revealed from satellite observations and its driving mechanism is attributed to the air-sea interaction^11^. The strengthening of the southwesterly accelerates the old offshore current and subsequent feedback of the cooled sea surface water to the atmosphere weakens winds above the sea surface^11^. The evolving processes comprise the enhancement of the dipole eddies through Rossby wave adjustment off Vietnam and the increase of static stability in the near sea surface atmosphere due to the cold sea surface water. However, the generation of the Rossby wave is not discussed in Ref.^11^. It is further noticed that the summer monsoon variability at periods 30–60 days could induce significant intraseasonal oscillations of sea level at periods 40–60 days in the SCS, particularly off the southeast coast of Vietnam^12,13^. The Rossby basin mode theory with the interaction of westward-propagating Rossby waves and eastward-propagating Rossby waves is used to explain this intraseasonal variation in the SCS circulation^12,13^.

To gain an insight into the boreal summer intraseasonal oscillation (BSISO) and associated ocean responses across the SCS, intensive field investigations have recently conducted using research vessel and moored instrument^14^. Since the summer southwesterly monsoon variability during the BSISO and its influence on the SCS circulation are not well-understood due to the lack of in situ observations, this study focuses on ocean responses to the summer monsoon oscillations in the central SCS. We analyze the velocity data collected by an acoustic Doppler current profiler (ADCP) deployed in the central-eastern SCS (see “Methods” section) together with concurrent satellite altimeter and wind data. We examine the dynamic linkage of intraseasonal ocean responses to the summer southwesterly monsoon oscillations using a numerical model. We also show the possibility of the Rossby waves propagating into the SCS from the east of the Philippines. The other potential mechanisms which may cause the ocean responses in intraseasonal timescale, such as the intrinsic basin modes and Rossby waves radiated from coastal Kelvin waves, are addressed.

## Results

### Intraseasonal oscillations in the zonal velocity

The 40–120 m depth-averaged ADCP velocity (*u*, *v*) and low-pass-filtered velocity (*u*_*l*_, *v*_*l*_) (see “Methods” section) are demonstrated in Fig. 1b,c. We focus on the most noticeable intraseasonal oscillations, particularly in *u*_*l*_ with amplitude ~ 0.1–0.2 m s^−1^. Note that the larger amplitude of *u*_*l*_ than *v*_*l*_ is presumably due to the zonal running isobaths around the ADCP mooring (lower panel in Fig. 1a), which guides the current to be zonally flowing, leading to a smaller amplitude in *v*_*l*_. The low-pass-filtered, satellite sea surface height-derived geostrophic velocity *u*_*g*_ and *v*_*g*_ (see “Methods” section) are overlaid on Fig. 1b,c (black curves) for comparison. Focusing on the summer months from May to November, the linear correlation coefficient of (*u*_*g*_, *v*_*g*_) and (*u*_*l*_, *v*_*l*_) is 0.61 for zonal and 0.45 for meridional component of velocity. Note that (*u*_*l*_, *v*_*l*_) does not represent the pure geostrophic current and thus there is certain discrepancy when compared with (*u*_*g*_, *v*_*g*_). In addition, the position of a SLA feature identified from the satellite altimeter data could have 0.25°–0.5° difference from the in-situ hydrography observations^15^. The aforementioned two factors create discrepancies between satellite (*u*_*g*_, *v*_*g*_) and in situ data-derived (*u*_*l*_, *v*_*l*_). Nevertheless, the observed *u*_*l*_ more or less follow the variability of satellite *u*_*g*_ during May–November (Fig. 1b).

It is easily seen that the time series of *u*_*l*_ in Fig. 1b exhibits four distinct quasi-regular oscillations at interval ~ 50 days during May–November when southwesterly monsoon prevailed in the SCS. These oscillations are similar to the 45-day interval atmospheric and oceanic intraseasonal events^11^. In addition to the 50-day oscillations, 14-, 70-, and 60-day oscillations with a mean velocity ~ 0.1 m s^−1^ were also observed but in winter. The low-pass-filtered temperature (Fig. 1d), although at 480 m depth, is likely varied with the oscillations of *u*_*l*_ as *u*_*l*_ turned from its peak eastward (westward) velocity to westward (eastward), the temperature increased (decreased). This correlation suggests that the ~ 50-day interval oscillations could reach 480 m.

**Figure 2:**
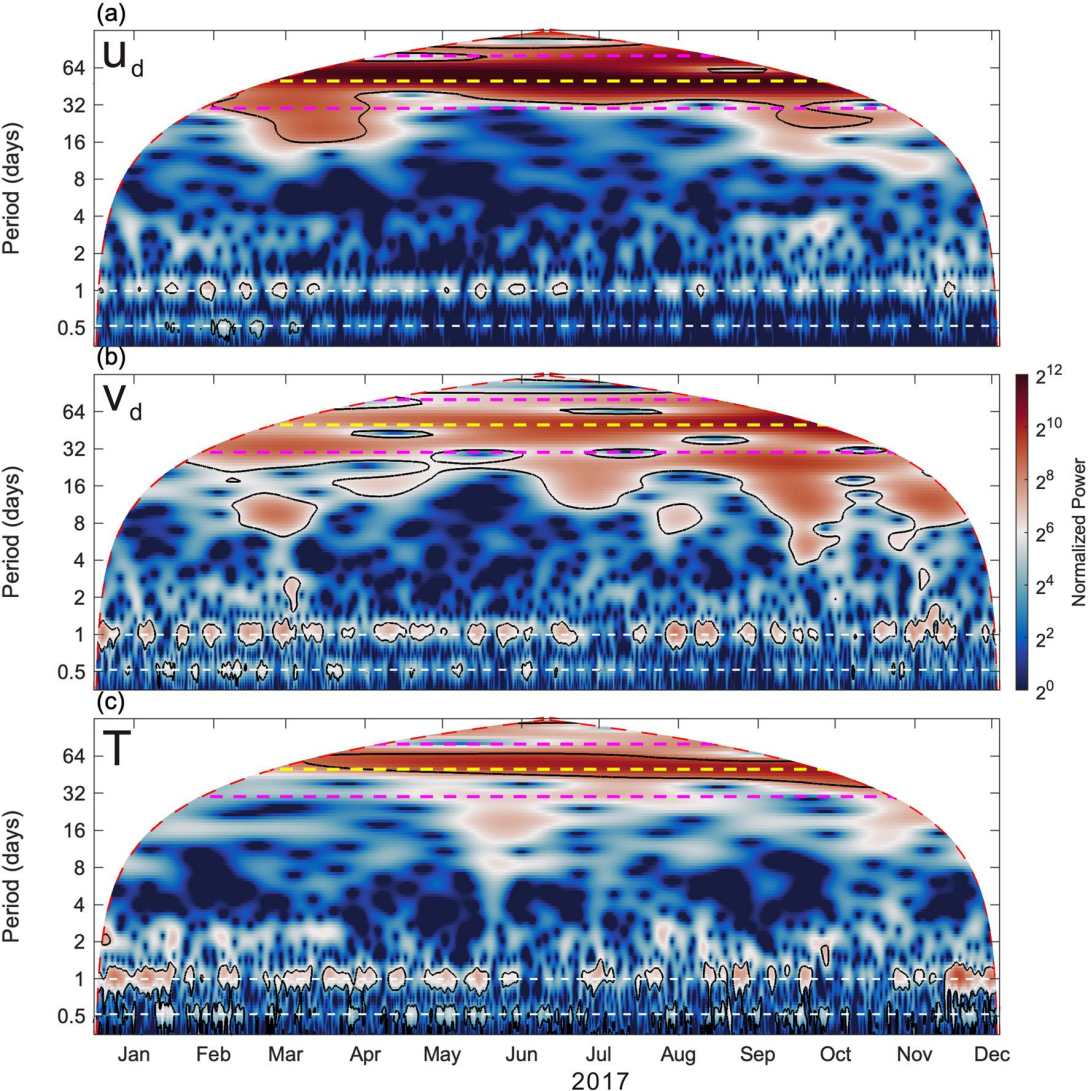
Wavelet power spectra of velocity and temperature. Wavelet spectra of depth-averaged velocity (**a**) *u*_*d*_, (**b**) *v*_*d*_, and (**c**) temperature obtained using a Morlet wavelet normalized with the corresponding variance. Black contours enclose the regions with a confidence level greater than 95%, red dashed lines indicate the cone of influence, white dashed lines represent the periods of diurnal K_1_ and semidiurnal M_2_ tides. Magenta lines indicate periods of 30- and 80-day, and the yellow dashed line in each panel indicates period 50-day.

To examine dominant frequencies of velocity oscillations in the upper 120 m layer, a Morlet wavelet analysis is applied to (*u*_*d*_, *v*_*d*_), and raw temperature, and the associated wavelet power spectra are plotted in Fig. 2. The corresponding period of the dominant spectral peaks is easily examined from Fig. 2. Leaving the obvious tidal energy aside, the clearest peak energy presents at period 50-day (yellow dashed lines in Fig. 2) during the southwesterly monsoon months enclosed by the two blue dashed lines in Fig. 1b–d.

### Comparison of velocity with satellite sea level anomaly and wind

**Figure 3:**
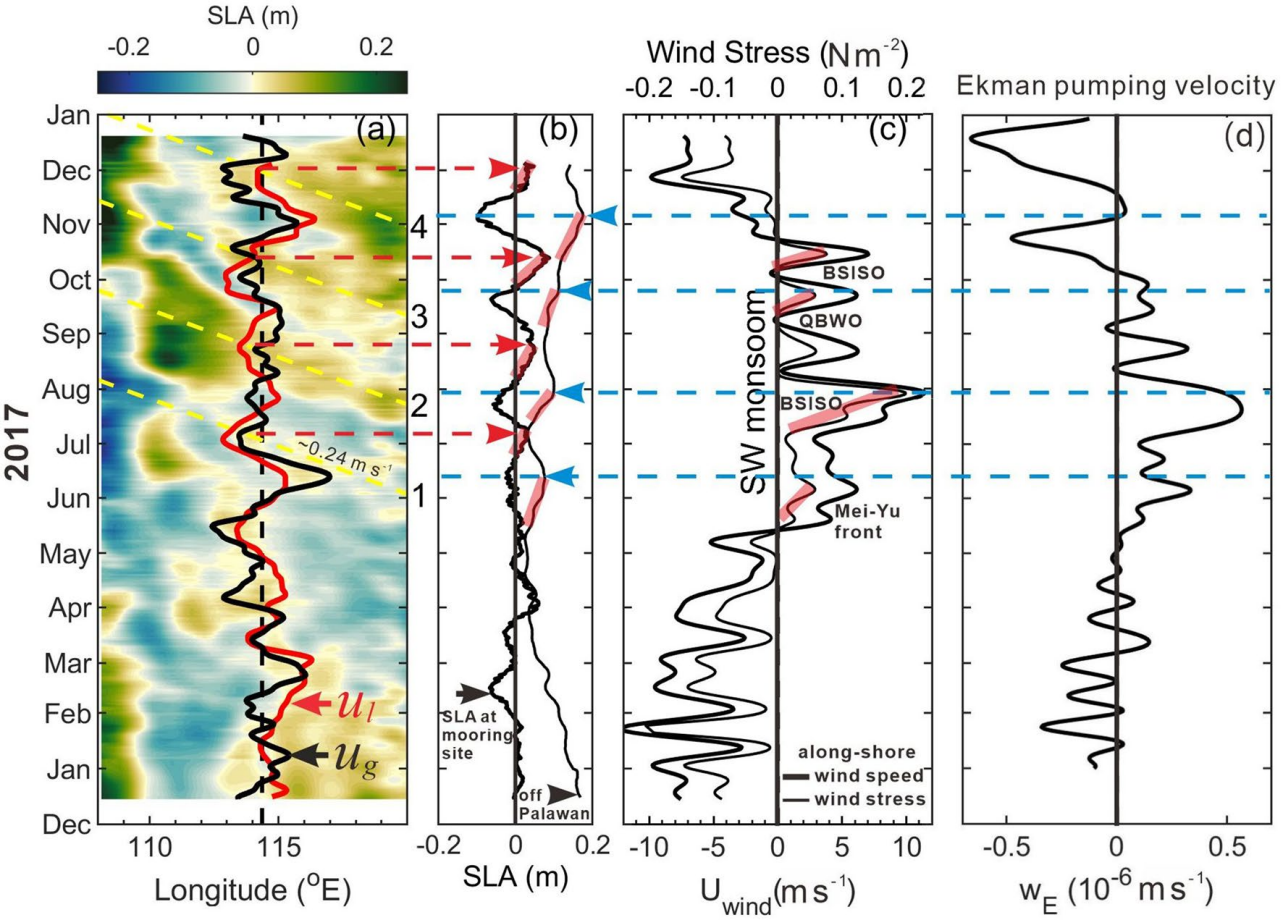
Satellite SLA and wind variation near the ADCP mooring. (**a**) Time versus longitude variation of the satellite SLA at 10.5° N. Black dashed line marks the longitude of the ADCP; red and black curves represent *u*_*l*_ and satellite altimeter data derived geostrophic velocity *u*_*g*_ (without scale), which are positive (negative) on the right (left) relative to the black dashed line. Yellow dashed lines indicate the westward-propagating SLA signals for four events marked by 1–4 on the right of the panel. (**b**) SLA near the mooring site (thick curve) and the area-averaged SLA (thin curve) in the polygon area enclosed by the red dashed lines off Palawan in Fig. 1a. (**c**) The low-pass-filtered, area-averaged alongshore CCMP wind speed (thick curve) and associated wind stress (thin curve). Red and blue dashed lines indicate timing of positive peak SLA near the mooring site and off the west coast of Palawan, respectively. Transparent red lines indicate rising stages of SLA in (**b**), and strengthening period of wind stress in (**c**) due to the four southwesterly monsoon oscillations marked in (**c**) where QBWO stands for quasi-biweekly oscillations. (**d**) The area-averaged, 8.33-day low-pass-filtered Ekman pumping velocity $\overline{{w_{E} }}$.

Figure 3a shows time versus longitude variation (i.e., Hovmöller diagram) of the satellite sea level anomaly (SLA) between 108° E and 120° E at 10.5° N overlaid with low-pass-filtered velocity *u*_*l*_ at the mooring longitude 114.3° E. The Hovmöller diagram is commonly used to trace the propagation of SLA signals, e.g., Rossby waves in the ocean^16^. The yellow dashed lines in Fig. 3a indicate slopes of the SLA signals, which are determined by connecting the positive peak SLA at the mooring longitude to its possible upstream source of the signal, i.e., off Palawan. The four intraseasonal events are marked by 1–4 on the right of Fig. 3a. The propagation speeds of the four SLA signals, i.e. the slopes of yellow dashed lines, are between 0.2 and 0.3 m s^−1^ with an average value of 0.24 m s^−1^ (toward the west), which are within the values derived from satellite altimeter data in the Pacific (0.25–0.35 m s^−1^)^16^ and consistent with the theoretical mode-1 baroclinic Rossby wave speed 0.25 m s^−1^ at 10.5° N^16–18^. Note that the SLA oscillations in Fig. 3a are similar to the composite SLA evolution shown in Fig. 7 of Ref.^11^ but the westward-moving Rossby waves speed 0.037 m s^−1^^11^ is apparently a typo of 0.37 m s^−1^. Also note that the phase speed of theoretical barotropic Rossby wave with wavelength O (1000 km) (or wavenumber *k* ~ 10^–6^ m^−1^) is approximately 0.4–0.5 m s^−1^, which is two times faster than that of mode-1 baroclinic waves.

The time-varying SLA near the ADCP mooring and SLA averaged over the polygon area off Palawan (red dashed polygon in Fig. 1a) are shown in Fig. 3b. The zonal and meridional components of CCMP winds (see “Methods” section) were rotated 44° clockwise to be the cross-shore and alongshore directions, respectively. Figure 3c shows the low-pass-filtered, area-averaged alongshore wind speed and corresponding wind stress. The peak SLA at the mooring site is indicated by red dashed arrow in Fig. 3a,b; the peak area-averaged SLA off Palawan is indicated by blue dashed arrow in Fig. 3b–d. Comparing Fig. 3b with a and c, the four peak SLA signals off Palawan lead the peak SLAs at the mooring location by ~ 25–30 days, and the peak SLAs off Palawan are visually correlated with the strengthening of the southwesterly (alongshore wind off Palawan) particularly for event 1, 2, and 3. We also compare the intraseasonal velocity variation with the SLA signals in Fig. 3b and alongshore winds in Fig. 3c during the boreal winter months, and find that the correlations between these variables are not as clear as those during the summer months. Examining the dynamics underlying these wintertime intraseasonal oscillations is beyond the scope of this study but merits a future study.

### Variability in the large-scale atmospheric forcing

The large-scale atmospheric forcing consists of four major southwest monsoon oscillations including two major BSISOs, a Mei-Yu front system, and quasi-biweekly oscillations (QBWO). The variations of the large-scale atmospheric forcing which likely caused these four southwesterly events marked in Fig. 3c are discussed in the Supplementary (Supplementary Discussion). The strengthened alongshore wind stress and corresponding increased shoreward Ekman transport most likely caused coastal SLA increase as indicated by the transparent bold red lines in Fig. 3b,c. Note, however, that SLA increase following event 4 in mid-October until early November was likely resulted from additional forcing as to be discussed later.

## Discussion

Mesoscale eddies are ubiquitous in the northern SCS^19,20^. By comparison, mesoscale eddies are less exhibited in the central than in the northern SCS according to the moored thermistor chains observations and statistics of satellite altimeter and hydrographic profile data^9,20^. Together with the daily satellite SLA images (not shown), the correlation between the intraseasonal velocity oscillations observed by the ADCP during May–November 2017 and eddy activities in the central SCS should be low. According to Fig. 3, the southwesterly monsoon oscillations and resultant coastal sea level pile-up and collapse are thus a plausible driving mechanism of the 50-day intraseasonal variations.

It is well-known that upper ocean responses to direct wind forcing comprise the generation of waves, currents, sea level variations, and turbulence at various temporal and spatial scales. The wind forcing could even induce intrinsic oscillation modes in an enclosed basin^12,13^. Barotropic or baroclinic waves at inertial or longer periods can further carry these ocean responses away from their formation region. With these possibilities in mind, the coastal sea level oscillations induced by the change of the alongshore wind-induced shoreward Ekman transport and Ekman pumping, and the resulting Rossby waves are hypothesized as the essential physical processes causing the intraseasonal oscillations observed by the ADCP.

**Figure 4:**
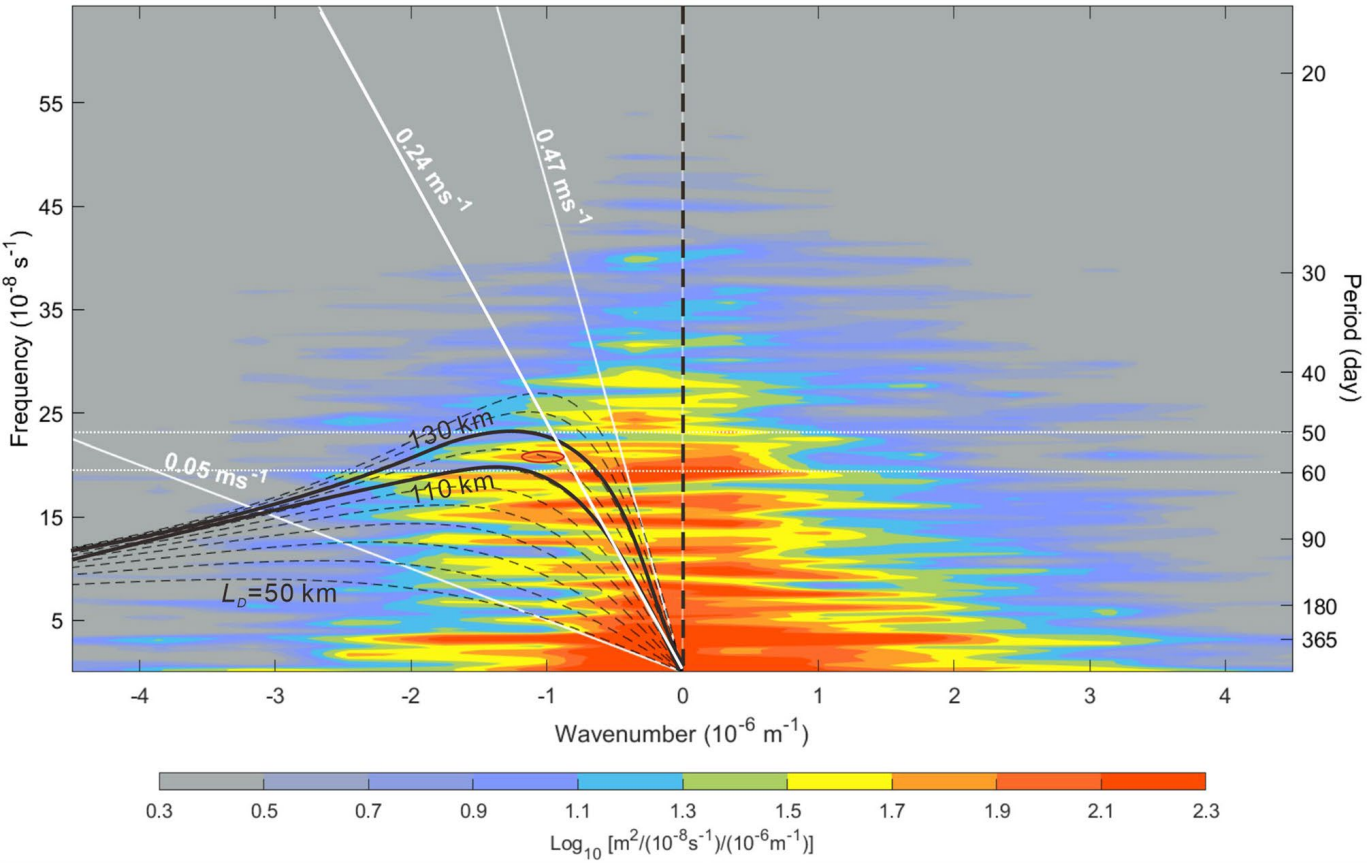
Frequency-wavenumber spectrum of satellite SLA. The frequency-wavenumber spectrum was calculated from a 17-year (2001–2017) satellite SLA dataset. The black dashed curves represent dispersion relations of Rossby waves calculated using the dispersion relation of Rossby wave at baroclinic Rossby deformation radius *L*_*D*_ from 50 to 150 km with an increment of 10 km. The two black thick curves mark dispersion relations at *L*_*D*_ = 110 and 130 km. The oblique white lines indicate phase speed of 0.05, 0.24, and 0.47 m s^–1^. The color shading indicates the energy density of the SLA oscillations. The red oval indicates westward-propagating energy density hump within periods 50 and 60 days.

To characterize Rossby waves in the central SCS, a frequency-wavenumber spectrum of SLA in the middle section of the SCS (Fig. 4) is obtained and discussed first. The spectrum was calculated from a 17-year (2001–2017) satellite SLA dataset obtained from CMEMS (see “Methods” section), which represents the temporal and spatial characteristics of SLA oscillations at 10.5° N across the central SCS from 108 to 120° E. In the westward-propagating SLA energy (i.e., negative wavenumber part), Fig. 4 shows that a relatively high energy density bulge toward the lower wavenumber within frequencies 5–25 × 10^−8^ s^–1^. The dispersion relation of Rossby waves (see “Methods” section) with the baroclinic Rossby deformation radius *L*_*D*_ from 50 to 150 km mostly covers this high energy density bulge. The wavenumber and frequency in a region within the dispersion relations at *L*_*D*_ = 110 and 130 km and periods between 50 and 60 days indicate that the wavelength for the energy density hump (red oval in Fig. 4) is ~ 1000 km and the phase speed is ~ 0.24 m s^−1^, which are the characteristics of the Rossby wave we regarded. Within periods 50 and 60 days, Fig. 4 also shows eastward-propagating SLA energy (positive wavenumber) with wavelength ~ 500 km shorter than that of the dominant westward-propagating SLA signals at this latitude. The eastward-propagating, shorter wavelength SLA signals are presumably reflection waves as westward-propagating Rossby waves impinge onto the Vietnam coast^12^. It is worth to note that a potential standing mode of SLA variability with the same wavelength and energy density in both propagating directions is found at period ~ 365 days (i.e. annual cycle) in Fig. 4, which deserves an in-depth study.

The aforementioned dynamics for the four atmosphere–ocean coupled intraseasonal events in the SCS is simple but surprisingly has not been reported in relevant publications. A similar event was observed off the Cape Verde Peninsula in the west African coast, where a Rossby wave emanating from the coast after a northerly alongshore wind burst was revealed by satellite altimeter data and ocean color images^21^. The northerly wind burst induced offshore Ekman drift is attributed to the driving mechanism of this westward-propagating Rossby wave^21^, which is similar to our dynamic interpretation for the intraseasonal oscillations. The physical processes we hypothesized are validated by an idealized, process-oriented numerical experiment described as follows.

**Figure 5:**
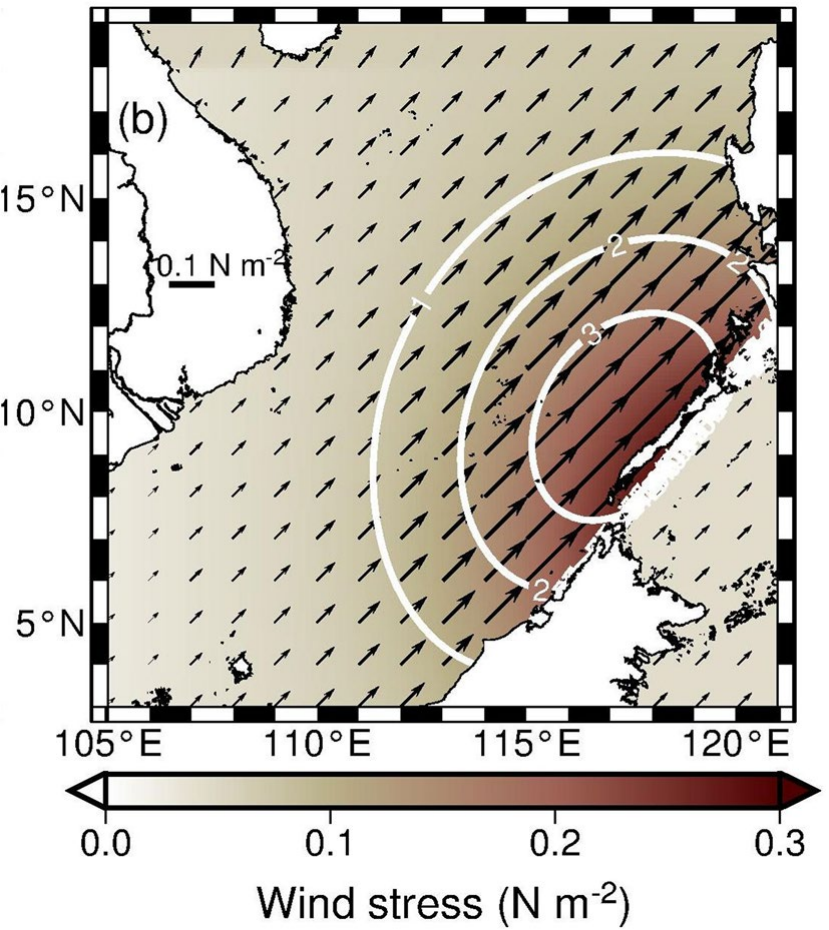
Spatial distribution of the maximum wind stress in the model. The length of each arrow represents the magnitude of wind stress. The white contours are constant values of wind stress curl (× 10^–7^ N m^–3^).

A three-dimensional, primitive equation model revised from the Princeton Ocean Model^22^ is used to simulate ocean responses to southwesterly monsoon oscillations in the SCS (see “Methods” section). To the southwest wind stress variation in Fig. 3c, a simplified single wind stress variation in a 50-day duration (see “Methods” section) is repeatedly applied to the SCS surface. The spatial distribution of the maximum wind stress during the 50-day interval is designed as Fig. 5. The peak alongshore wind stress is aligned along Palawan and the maximum wind stress curl is located off the west coast of Palawan (Fig. 5). The integration of a numerical experiment is 500 model days (or 10 repeated cycles of wind stress variation) and results during model day 150 to 360 are selected to analyze. Note that the modeling strategy is simply to produce the hypothesized physical processes instead of to realistically simulate details of wind variations and the ADCP observed velocity.

**Figure 6:**
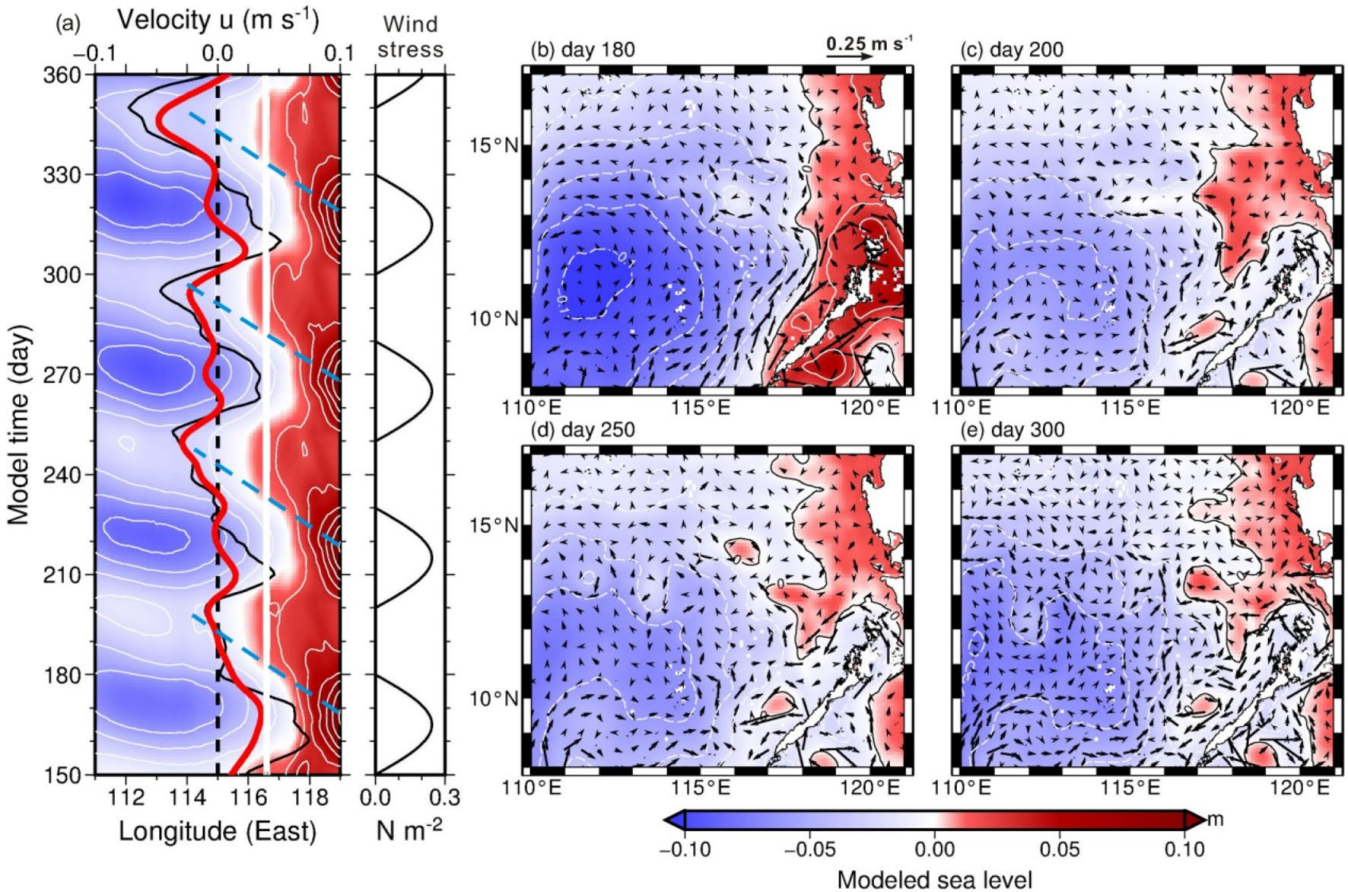
Model produced sea level and velocity variations from day 150 to 360. (**a**) Time versus longitude of sea level at 10.5° N and wind stress near the coast of Palawan at (118° E, 10.5° N). The 9-day low-pass-filtered 25 m depth zonal velocity (black curve) and upper 120 m depth-averaged zonal velocity (red curve) at (115° E, 10.5° N) are overlaid on the SLA plot. Blue dashed lines in (**a**) are determined by the same criterion as the yellow dashed lines in Fig. 3a, which indicate the slope of the high sea level signals. The sea level (colored) and 25 m depth velocity (arrows) at model day (**b**) 180, (**c**) 200, (**d**) 250, and (**e**) 300. The sea level contour interval is 0.02 m.

**Figure 7:**
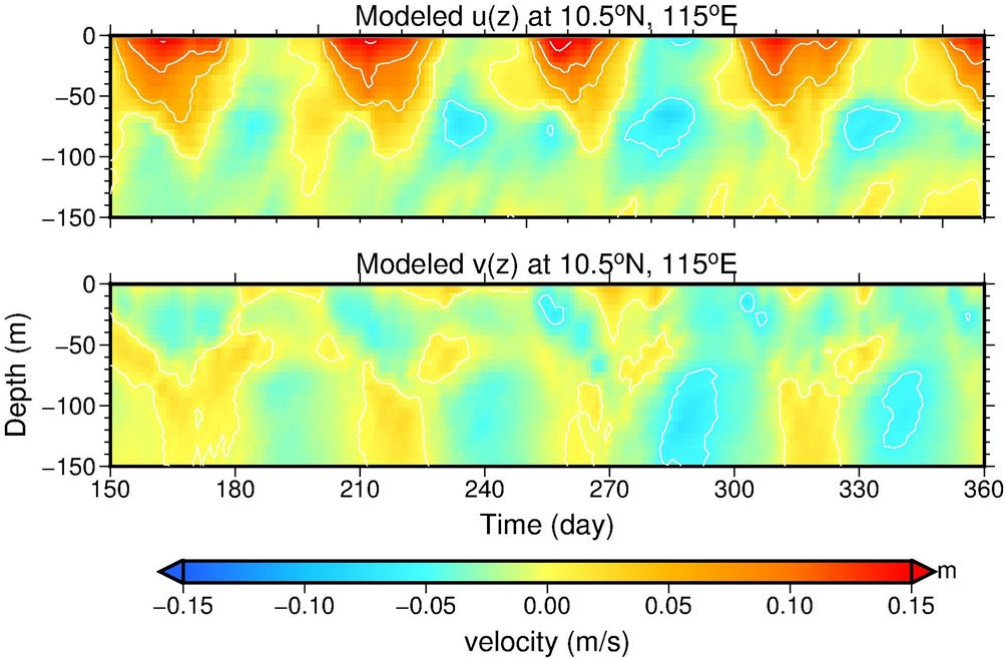
Model produced velocity profiles at the mooring site. The upper 150 m velocity of u (upper panel) and v (lower panel) at grid (115° E, 10.5° N) where is close to the ADCP mooring location.

During the model day 150 to 360, the time versus longitude (111–119° E) variation of model-produced sea level at 10.5° N in Fig. 6a shows that sea level varies with the four simplified alongshore wind stress oscillations in Fig. 6b at model grid (118.0° E, 10.5° N) west of Palawan. The time-varying alongshore winds cause ~ 0.1 m sea level difference near the west coast of Palawan, and westward-propagating sea level signals can be seen from the Hovmöller diagram in Fig. 6a. Results from the numerical experiment qualitatively capture the characteristics of SLA variations in Fig. 3a. The model-produced zonal and meridional velocity profiles in the upper 150 m at grid (115° E, 10.5° N) present the vertical structure of velocity and the relationship between the two velocity components (Fig. 7), which are consistent with the essential characteristics obtained from the observations (Fig. 1b,c). The velocity at 25 m (black curve in Fig. 6a) and upper 120 m depth-averaged, 9-day low-pass filtered velocity (red curve in Fig. 6a), although smaller than the observations, resemble the variation of the observed velocity (red curve in Fig. 3a).

From a broader view of the sea level distribution, Fig. 6b shows heaved coastal sea levels along the eastern boundary of the SCS at day 180 (wind stress just stopped). At day 200, the beginning of next wind burst (Fig. 6c), dome-like sea level distributions, e.g., centered at (117.3° E, 10.5° N) and near Palawan and Luzon Island between 117.5–119° E and 11–15° N, are presumably generated from the collapsed coastal sea level high 20 days after previous wind strengthening event. These sea level domes propagate westward but could be distorted and even dissipated, particularly west of 114–115° E, by the next wind strengthening event in the model during their westward propagation. This may explain that why satellite SLA signals became blurred roughly west of 113° E (Fig. 3a). The formation, propagation, and associated modification of these propagating sea level signals accompanied with anticyclonic circular currents are reproduced during each repeated wind strengthening event (Fig. 6d,e for day 250 and 300, respectively).

Each high sea level signal as indicated by the blue dashed lines in Fig. 6a, determined as the criterion of the yellow dashed lines in Fig. 3a, propagates westward at a speed of ~ 0.21 m s^–1^ east of 114° N. The zonal component of depth-averaged velocity is westward with peak velocity ~ 0.05 m s^–1^ when the relatively high sea level signal arrives at the modeled ADCP, and turns to be eastward (~ 0.04 m s^–1^) when the sea level decreases. The resemblance between the characteristics obtained from the model results and from the ADCP and satellite observations lends support to our hypothesis.

Conceivably, the increase of alongshore wind stress-induced shoreward Ekman transport results in the coastal sea level rise along the west coast of Palawan. The wind stress curl can additionally contribute to the coastal sea level variation. A positive wind stress curl causes positive Ekman pumping velocity *w*_*E*_ (i.e., upwelling), which uplifts thermocline producing pressure gradients and consequently creates cyclonic circulation through geostrophic balance^23^. Therefore the relatively higher sea level on the right hand side when facing the direction of the geostrophic current, i.e. around the cyclone, aids to coastal sea level rise off the west coast of Palawan. When the southwesterly monsoon was strengthened along Palawan, the wind stress curl was positive west of Palawan as indicated by the positive Ekman pumping velocity $\overline{{w_{E} }}$ (see “Methods” section and Fig. 3d). We estimate that, as the radius of a wind stress curl-created cyclone is O(500 km) (larger than half width of the SCS) and its typical mean orbital velocity *V*_*r*_ is O(0.1 m s^−1^) (see currents in the offshore side of Palawan in Fig. 6b), the sea level difference between the cyclone’s peripheral and center could be 0.13 m, under the geostrophic balance in the radial direction (*r*): *fV*_*r*_ = $g\frac{{\partial \eta }}{{\partial r}}$, where *η* is sea level, *f* is the Coriolis parameter (= 2.67 $\times$ 10^−5^ s^−1^ at 10.5° N), and *g* (= 9.8 m s^−2^) is the gravitational acceleration. This wind stress curl induced coastal sea level rise could be 0.065 m from an equilibrium sea level in terms of mass conservation of this cyclone and a linear variation of the sea level difference, which accounts for approximately 30–65% of the SLA off Palawan. The relationship between the Ekman pumping velocity and the coastal sea level rise off Palawan is examined from Fig. 3b,d. During May through November, when the alongshore wind and associated $\overline{{w_{E} }}$ strengthened (Fig. 3c,d), the area-averaged SLA off Palawan increased (Fig. 3b). When the alongshore wind and $\overline{{w_{E} }}$ subsequently weakened, the dome-like SLA was unleashed off Palawan. Comparing Fig. 3b with d, the increase of $\overline{{w_{E} }}$ led the peak area-averaged SLA by ~ 10-day for event 1–3 and was almost in phase for event 4.

Note that the low frequency temperature variation (red line in Fig. 1d) is also correlated with the change of $\overline{{w_{E} }}$ (Fig. 3d). It suggests that, as $\overline{{w_{E} }}$ increased, the temperature decreased, and vice versa. The increase (decrease) of the low frequency variation of temperature, although measured at 480 m, suggests that the thermocline was depressed (heaved) in the central eastern SCS.

**Figure 8:**
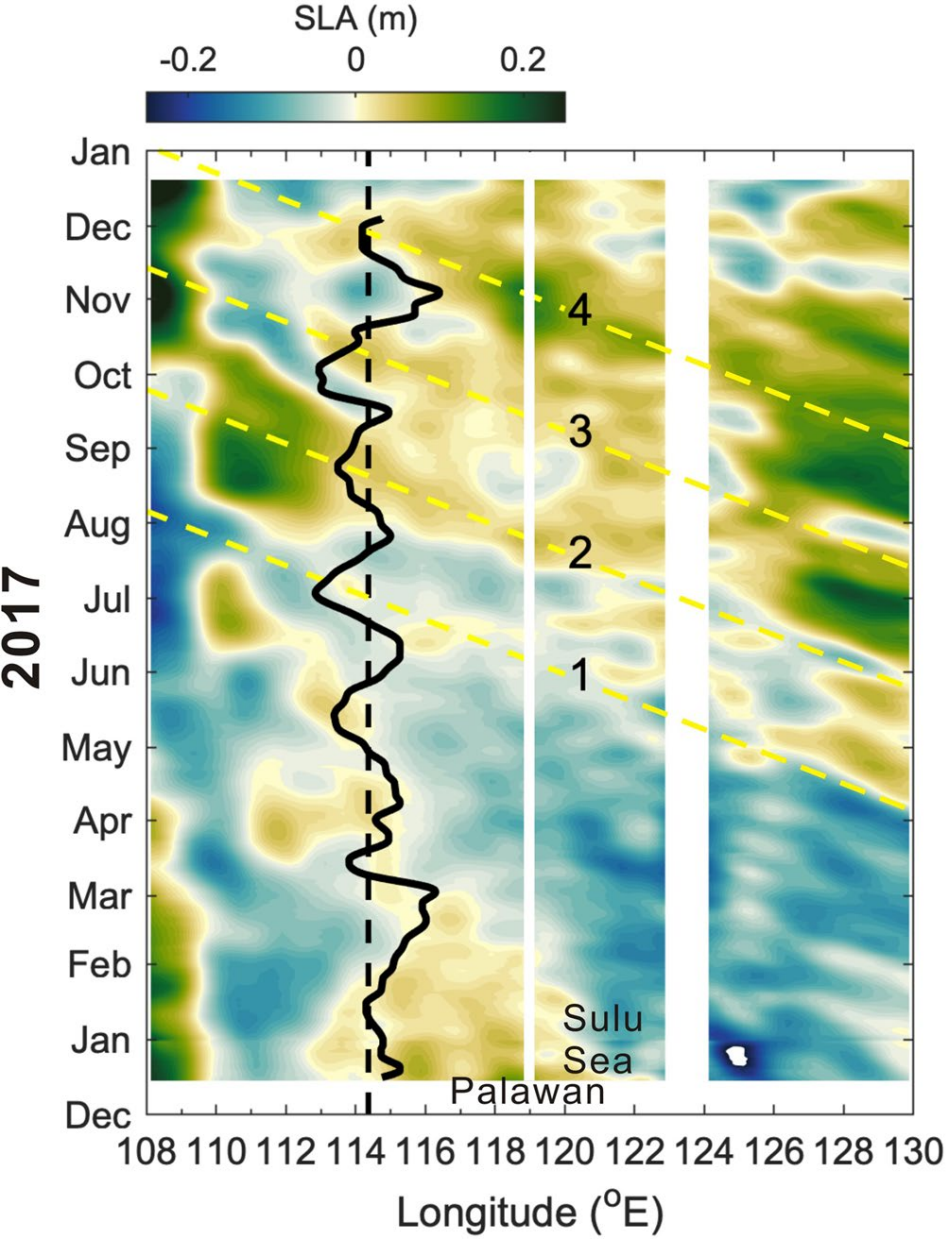
Time versus extended longitude SLA variation of Fig. 3a. Same as Fig. 3a but the longitude is extended to 130° E to include the tropical North Pacific east of the Philippine archipelago.

Since oceanic Rossby waves are large-scale activity with wavelengths of a few hundred to thousands kilometers in the open ocean^16^, their remote influence from the Pacific east of the Philippines to the central SCS may not be excluded. In this light, we further discuss if westward-propagating Rossby waves off the east coast of the Philippines can influence the SLA west of Palawan. The Hovmöller diagram of satellite SLA is extended to 130° E (Fig. 8) for the evaluation of this possibility. The four yellow dashed lines in Fig. 8 suggest that the coastal SLA increase of event 1–3 are likely not directly correlated to those westward-propagating SLA signals east of the Philippines. Figure 8 suggests that the most possible penetration of remote Rossby waves from the east of the Philippines to the central SCS is correlated with event 4. The energy of sizable SLA signals (> 0.2 m) which reached east of the Philippines during September–October (Fig. 8) could leak through islands of the eastern Philippines into the Sulu Sea and generate ocean activities there. The associated increase of SLA energy in the Sulu Sea could further leak into the central SCS through the two major passages connecting the Sulu Sea to the SCS and contributed to the coastal SLA increase off the west coast of Palawan for event 4. However, the possibility needs a thoughtful validation in the future.

The Rossby wave could also be generated by the low-frequency, poleward-propagating baroclinic coastal Kelvin waves in the midlatitude ocean^24,25^. As a coastal Kelvin wave propagates poleward, the Coriolis force increases and consequently the Kelvin wave is enhanced in amplitude, which in turn causes energy leakage to create Rossby wave emanating westward from the eastern boundary^24,25^. Moreover, the Rossby basin mode^11,12^ (or standing wave modes^26^) in the deep SCS basin may be the contribution mechanism of the intraseasonal velocity oscillations in the central SCS. The standing mode and the associated nodal point or propagating mode across the central SCS need to be carefully examined from more comprehensive in situ and satellite observations. Whether or not these mechanisms could exist along the eastern boundary of the low-latitude central SCS merits a future study as well.

## Conclusion

Here we analyze atmospheric and oceanic oscillations and their dynamic relationship in the central SCS during the boreal summer 2017. Four coupled oscillation events at intraseasonal timescale ~ 50 days are revealed from the ADCP observations together with satellite wind and altimeter data between May and November 2017. Each event comprises southwesterly monsoon strengthening/weakening, associated coastal SLA accumulation/collapse via Ekman transport and Ekman pumping west of Palawan, and consequent westward-propagating Rossby wave in the central SCS. The resultant Rossby wave passing through the ADCP mooring causes distinct velocity oscillations, particularly in the zonal component of velocity. The satellite SLA-derived Rossby wave speeds are 0.2–0.3 m s^−1^, which are in a reasonable range around the theoretical phase speed of Rossby waves 0.25 m s^–1^ at 10.5° N. The evolving processes are examined by an idealized numerical model. Results from the numerical experiment with simplified wind forcing resemble the essential characteristics analyzed from the ADCP measured velocity and satellite altimeter observations, lending support to our dynamical interpretation of the intraseasonal velocity oscillations. This also explains the origin of the Rossby waves observed in the central SCS previously^11^.

Note that the SLA changes in the northwestern Pacific and Sulu Sea following the southwesterly monsoon oscillations suggest other possible influences. One is that the Sulu Sea could amplify the coastal SLA variability off Palawan. The other is that Rossby waves with sufficient strong SLA (> 0.2 m) east of the Philippine may penetrate the Mindanao Current, islands, and the two passages north and south of Palawan into the central SCS. Together with the Rossby basin mode^12,13^ (i.e., the seiche modes in the SCS deep basin^26^) and Rossby waves radiated from low-frequency baroclinic coastal Kelvin waves^24,25^, these potential driving mechanisms for the intraseasonal velocity oscillations during summer monsoon season in the central SCS warrant future observational and modeling studies.

## Methods

### Moored ADCP observations

To improve the observational database during interactions between the BSISO and large-scale ocean–atmosphere circulation and the associated responses of SCS, joint ocean–atmosphere observations were conducted in 2016 and 2017 under the project South China Sea Two-Island Monsoon Experiment (SCSTIMX)^14^. The observational group of SCSTIMX conducted underway measurements onboard R/V Ocean Researcher I while sailing from Taiwan to Taiping Island (Fig. 1a) and back in the SCS. A 75-kHz ADCP was moored at 114.3° E and 10.5° N on 18 December 2016 and was recovered on 18 December 2017. The depth at the mooring site is 2028 m, and the isobaths around the mooring are approximately in the zonal direction (Fig. 1a). The ADCP was fixed at a depth of 480 m and measured the velocity profile upward from ~ 470 to 40 m with a vertical resolution of 8 m (i.e., bin size). The thermometer in the ADCP measured the temperature at 480 m every 10 min.

The magnitude of velocity suggests that significant variations in the zonal and meridional velocity (*u* and *v*) occurred mostly in the upper 150 m, and therefore *u* and *v* in the upper 40 − 120 m were vertically averaged as *u*_*d*_ and *v*_*d*_ (gray curves in Fig. 1b,c) for analysis. To retain intraseasonal variations, *u*_*d*_ and *v*_*d*_ were low-pass filtered as *u*_*l*_ and *v*_*l*_ (red curves in Fig. 1b,c) using the Butterworth filter with a cutoff frequency of 5 $\times$ 10^−3^ cycles per hour (cph) (equivalent to a period of 8.33 days). The inertial and tidal oscillations were excluded from the velocity data. The raw temperature was also low-pass filtered (red curve in Fig. 1d).

### Satellite remote-sensing data

To facilitate data analysis, 0.25° resolution, satellite-altimeter-derived daily SLA and geostrophic velocity data were obtained from the Copernicus Marine Environment Monitoring Service (CMEMS) at https://resources.marine.copernicus.eu. The satellite geostrophic velocity was 8.33-day low pass filtered (*u*_*g*_). The 0.25° gridded vector winds (10 m above sea surface) produced by the Cross-Calibrated Multi-Platform (CCMP) version 2.0^27^ were obtained at http://www.remss.com (see the supporting information for how the CCMP winds are produced). The 850 hPa wind data were obtained from the National Centers for Environmental Prediction (NCEP) Final Analysis (FNL) Operational Model Global Tropospheric Analyses, continuing from July 1999, at https://rda.ucar.edu/datasets/ds083.2/ to interpret the large-scale atmospheric background (see Supplementary Discussion). The six hourly CCMP and NCEP-FNL wind data were daily averaged in our analysis.

### Calculation of frequency-wavenumber spectrum of SLA

A 17-year (2001–2017) longitude (108–120° E) daily SLA dataset was obtained from the CMEMS at https://resources.marine.copernicus.eu. The 0.25° resolution SLA was meridionally averaged over 10 to 11° N and the 108–120° E, meridionally-averaged SLA time series was used to calculate the frequency-wavenumber spectrum. The dispersion relation of Rossby waves in Fig. 4 was calculated using the equation^28^:

1 $$ {{\upomega }} =  - \frac{{\beta k}}{{k^{2}  + l^{2}  + 1/L_{D} ^{2} }}, $$

where *k* and *l* are wavenumbers in the zonal and meridional directions, respectively, and *L*_*D*_ is the mode-1 baroclinic Rossby radius of deformation. The Rossby parameter *β* (= 2Ω cos *φ a*^−1^, where Ω is the angular velocity of the Earth’s rotation, *φ* the latitude, and *a* the Earth’s radius) is 2.24 $\times$ 10^−11^ s^−1^ m^−1^ at 10.5° N. The Rossby radius of deformation for the calculation of the dispersion relation in Eq. (1) was given from 50 to 150 km with an interval of 10 km (Fig. 4).

### Numerical model

The model is essentially the same as the one used in Ref.^29^ except that it is driven by only idealized southwesterly oscillation over the SCS. The model is a three-dimensional, non-linear, primitive equation model with Boussinesq and hydrostatic approximations. The governing equations consist of momentum balance, temperature and salinity, and continuity equations. The model domain covers a broad range within 100° E–135° W and 2–40° N; the horizontal grid spacing is 0.1° between 2–40° N and west of 145° E in the zonal and meridional directions and 0.2° east of 145° E in the zonal direction. There are 32 uneven σ–layers in the vertical. The center of each vertical grid in the *σ*-coordinate is − 0.001, − 0.003, − 0.006, − 0.008, − 0.011, − 0.016, − 0.020, − 0.025, − 0.030, − 0.034, − 0.039, − 0.043, − 0.048, − 0.052, − 0.057, − 0.061, − 0.066, − 0.070, − 0.075, − 0.080, − 0.084, − 0.089, − 0.097, − 0.108, − 0.125, − 0.148, − 0.182, − 0.261, − 0.375, − 0.489, − 0.659, and − 0.886 respectively from the top to the bottom layer. Model topography is established using 2-min Gridded Global Relief Data (ETOPO2v2) (http://www.ngdc.noaa.gov/mgg/global/etopo2.html). The topography is flattened at 6000 m for depths greater than 6000 m.

The model ocean is initially motionless and the density field is horizontal homogeneous and vertically stratified with an idealized temperature profile. Initial temperature field is horizontal homogeneous and vertically stratified by setting temperature asT(z) = 2 + 25 exp(z/1000), where z is depth^29^. Salinity is 34.5 throughout the numerical integration. To reproduce the intraseasonal oscillation of southwest wind, a 50-day duration of simplified southwesterly monsoon variation is applied to the sea surface in the SCS after the onset of the model integration. The wind stress is increased and decreased as a half sine function (0–π) from day 1 to 30 and remains 0 from day 31 to 50. The zonal and meridional wind stress, *τ*_*x*_ and *τ*_*y*_, are calculated by

$$ \tau _{x} \left( {{\text{x}},{\text{ y}},{\text{ t}}} \right){\text{ }} = {\text{ }}0.{\text{3 sin}}\left( {{\text{t }}\pi /{\text{3}}0} \right){\text{ exp}}\left[ { - {\text{ }}\left( {y - {\text{7}}} \right)^{{\text{2}}} /{\text{36}}} \right]{\text{ sin}}\left( {x\pi /{\text{2}}/{\text{17}}} \right){\text{ N m}}^{{ - {\text{2}}}} ,{\text{ and}} $$

$$ \tau _{y} \left( {{\text{x}},{\text{ y}},{\text{ t}}} \right){\text{ }} = \tau _{x} \left( {{\text{x}},{\text{ y}},{\text{ t}}} \right), $$

where t is time in days (from day 0 to day 30), *x* and *y* are longitude and latitude in degrees, respectively. The heat flux is zero at the sea surface. The time-varying wind stress is repeated during the numerical integration every 50 days until day 500, and the integration of a model run is 540 days. The integration time steps are 15 s and 600 s for the external and internal modes, respectively. Oceanic oscillations in the model are spun-up approximately after the first 120 days of numerical integration. The model results during four consecutive wind burst events from day 150 to 360 are presented and discussed.

### Calculation of the Ekman pumping velocity

The Ekman pumping velocity $$w_{E}$$ is estimated using the CCMP daily winds in a polygon region off Palawan (enclosed by red dashed lines in Fig. 1a) through $$w_{E}$$ =  − *ρ*_0_^−1f−1^ $$\nabla  \times \overset{\lower0.5em\hbox{$\smash{\scriptscriptstyle\rightharpoonup}$}} {\tau }$$, where $$\overset{\lower0.5em\hbox{$\smash{\scriptscriptstyle\rightharpoonup}$}} {\tau }$$ is wind stress calculated using $$\overset{\lower0.5em\hbox{$\smash{\scriptscriptstyle\rightharpoonup}$}} {\tau }  = \rho _{a} C_{D} \overset{\lower0.5em\hbox{$\smash{\scriptscriptstyle\rightharpoonup}$}} {U} _{{10}}^{2}$$ with air density *ρ*_*a*_ = 1.2 kg m^−3^, drag coefficient *C*_*D*_ = 1.3 × 10^−3^, wind velocity at 10 m above the sea surface ($$\overset{\lower0.5em\hbox{$\smash{\scriptscriptstyle\rightharpoonup}$}} {U} _{{10}}$$), reference seawater density *ρ*_0_ = 1026 kg m^−3^, the Coriolis parameter *f* = 2.67 $$\times$$ 10^−5^ s^−1^ at 10.5° N, and operator $$\nabla  = \frac{\partial }{{\partial {\text{x}}}}\overset{\lower0.5em\hbox{$\smash{\scriptscriptstyle\rightharpoonup}$}} {i}  + \frac{\partial }{{\partial {\text{y}}}}\overset{\lower0.5em\hbox{$\smash{\scriptscriptstyle\rightharpoonup}$}} {j}$$. The estimated pumping velocities are further averaged over the polygon area off Palawan ($$\overline{{w_{E} }}$$). A 8.33-day low-pass filter is applied to $$\overline{{w_{E} }}$$ to retain the intraseasonal variation (Fig. 3d).

## Supplementary Information

Supplementary Information.

## Data Availability

Satellite SLA and geostrophic velocity were obtained from CMEMS (https://resources.marine.copernicus.eu/?option=com_csw&view=details&product_id=SEALEVEL_GLO_PHY_L4_REP_OBSERVATIONS_008_047). The CCMP Version-2.0 wind analyses are produced by RSS (http://www.remss.com/support/data-shortcut/). NCEP FNL data are available at https://rda.ucar.edu/datasets/ds083.2/. The BSISO indices were downloaded from http://iprc.soest.hawaii.edu/users/kazuyosh/Bimodal_ISO.html. The ADCP dataset is available at 10.5281/zenodo.4032136.

## Code availability

The numerical model code for this study is available at 10.17632/39r3gzdwcj.1.
