# Supplementary material for: Mooring observed intraseasonal oscillations in the central South China Sea during summer monsoon season
Source: Sci Rep. 2021 Jul 1;11:13685. doi: 10.1038/s41598-021-93219-3 (PMC8249644; doi:10.1038/s41598-021-93219-3)
Supplement: Supplementary file 1 — Supplementary Information. [file 41598_2021_93219_MOESM1_ESM.pdf]

Supplementary Information for

Mooring observed intraseasonal oscillations in the central South China Sea during summer monsoon season

Sen Jan[1], Ming-Huei Chang[1], Yiing Jang Yang[1], Chung-Hsiung Sui[2], Yu-Hsin Cheng[1], Yu-Yu Yeh[1], and Chung-Wei Lee[2]

[1]Institute of Oceanography, National Taiwan University, Taipei, Taiwan
[2]Department of Atmospheric Sciences, National Taiwan University, Taipei, Taiwan

Correspondence to: senjan@ntu.edu.tw

Supplementary Discussion

Analysis of monsoon oscillations over the South China Sea during 2017

To analyze the large-scale atmospheric circulation, we used two sets of wind fields: the Cross-Calibrated Multi-Platform (CCMP)[1] (http://www.remss.com) and the National Centers for Environmental Prediction (NCEP)[2] Final Analysis (FNL) [https://rda.ucar.edu/datasets/ds083.2/]. The CCMP wind product combines version-7 Remote Sensing Systems (RSS) radiometer wind speeds, Quick Scatterometer (QuikSCAT) and Advanced Scatterometer (ASCAT) wind vectors, moored buoy wind data, and the European Centre for Medium-Range Weather Forecasts Reanalysis–Interim (ERA–Interim) model wind fields using a variational analysis method. Both data are of six hourly resolution but averaged to daily and also averaged in space to 1.0° resolution in this study.

Judging from pentad maps of OLR and 850hPa winds[3] during the period of May–November and the phase diagram of BSISO in Supplementary Figure 1, we identify four major Southwesterly monsoon oscillations shown in Supplementary Figures 2-4. The four SW events occurred during the following periods: mid-May to early June (SW1), mid-July to early August (SW2), two pentads centered around 13 and 23 September (SW3), and 2-3 pentads centered around mid-October (SW4). The overall evolution of the four SW events are examined below.

For SW1, the southwesterly increased with a front approaching the South China Sea (SCS) (Supplementary Figures 2a–c) and decreased as the front dissipated (Supplementary Figures 2e–f). The upstream westerly over eastern Indian Ocean was also boosted by a cyclone pair (Supplementary Figures 2c). In SW2, the westerly over the SCS was accelerated by an enhanced (suppressed) convection in the north (south) (Supplementary Figures 3b, c). The convection dipole elongated southeastward from Bay of Bengal to eastern Maritime Continent (Supplementary Figures 3b, c) and propagated northeastward (Supplementary Figures 3d, e). Thereafter the convection phase reversed in the following pentads, with enhanced (suppressed) phase in the southern (northern) SCS inducing the easterly anomaly (Supplementary Figures 3e–f). In SW3, the BSISO amplitude (Supplementary Figure 1) was slightly less than the unit circle; whereas the propagation was clear on the phase chart (Supplementary Figure 1c). An enhanced convection appeared over the equatorial eastern Indian Ocean in the first pentad (Supplementary Figure 4a), propagated northeastward into the SCS and accelerated southwesterly three pentads later (Supplementary Figure 4d). It dissipated and was replaced by a suppressed phase in the following pentads

(Supplementary Figures 4e, f), while the wind anomaly turned easterly. A higher-frequency variation also caused the Outgoing Long-wave Radiation (OLR) anomaly in the SCS changing sign almost every pentad in SW3, which might be quasi-biweekly oscillation (QBWO) that propagated northwestward (Supplementary Figures 4a–e). In SW4, the enhanced convection developed over eastern Indian Ocean in Pentad 1 and 2 (Supplementary Figures 5a–b). After it propagated northeastward to the northwestern Pacific in the following pentads (Supplementary Figures 5c–e), several tropical storm geneses occurred, including typhoon Khanun and Lan; their broad low-level cyclonic circulation induced strong westerly anomaly in the SCS.

In summary, SW2 and SW4 were associated with major BSISOs, SW1 was presumably caused by a Mei-Yu front (also called plum rain) in the East Asian rainy season, and SW3 was influenced primarily by northwestward-moving QBWOs.

We noticed that the Rossby wave signals observed in the satellite SLA exist mostly in summer instead of winter in the central SCS. Presumably, this seasonal difference can be attributed to eastward-moving intraseasonal oscillations (ISOs) being associated with northward propagation in boreal summer. In wintertime, eastward-moving ISOs detour slightly southward around the Maritime Continent islands and thus influence the SCS circulation less in winter than in summer. To verify our inference, further in situ observations together with a modeling approach are necessary for a more quantitative study of atmosphere and ocean feedback processes.

Supplementary References

1. Wentz, F.J., Scott, J., Hoffman, R., Leidner, M., Atlas, R., & Ardizzone, J. Remote Sensing Systems Cross-Calibrated Multi-Platform (CCMP) 6-hourly ocean vector wind analysis product on 0.25 deg grid, Version 2.0. Remote Sensing Systems, Santa Rosa, CA. (2015). Available online at www.remss.com/measurements/ccmp. Accessed 13 Aug 2018.
2. National Centers for Environmental Prediction/National Weather Service/NOAA/U.S. Department of Commerce, updated daily. NCEP FNL Operational Model Global Tropospheric Analyses, continuing from July 1999. Research Data Archive at the National Center for Atmospheric Research, Computational and Information Systems Laboratory. (2000). https://doi.org/10.5065/D6M043C6. Accessed 08 Jul 2020.

3. Kikuchi, K., Wang, B., & Kajikawa, Y. Bimodal representation of the tropical intraseasonal oscillation. Clim. Dyn., 38, 1989-2000. (2012). doi:10.1007/s00382-011-1159-1.

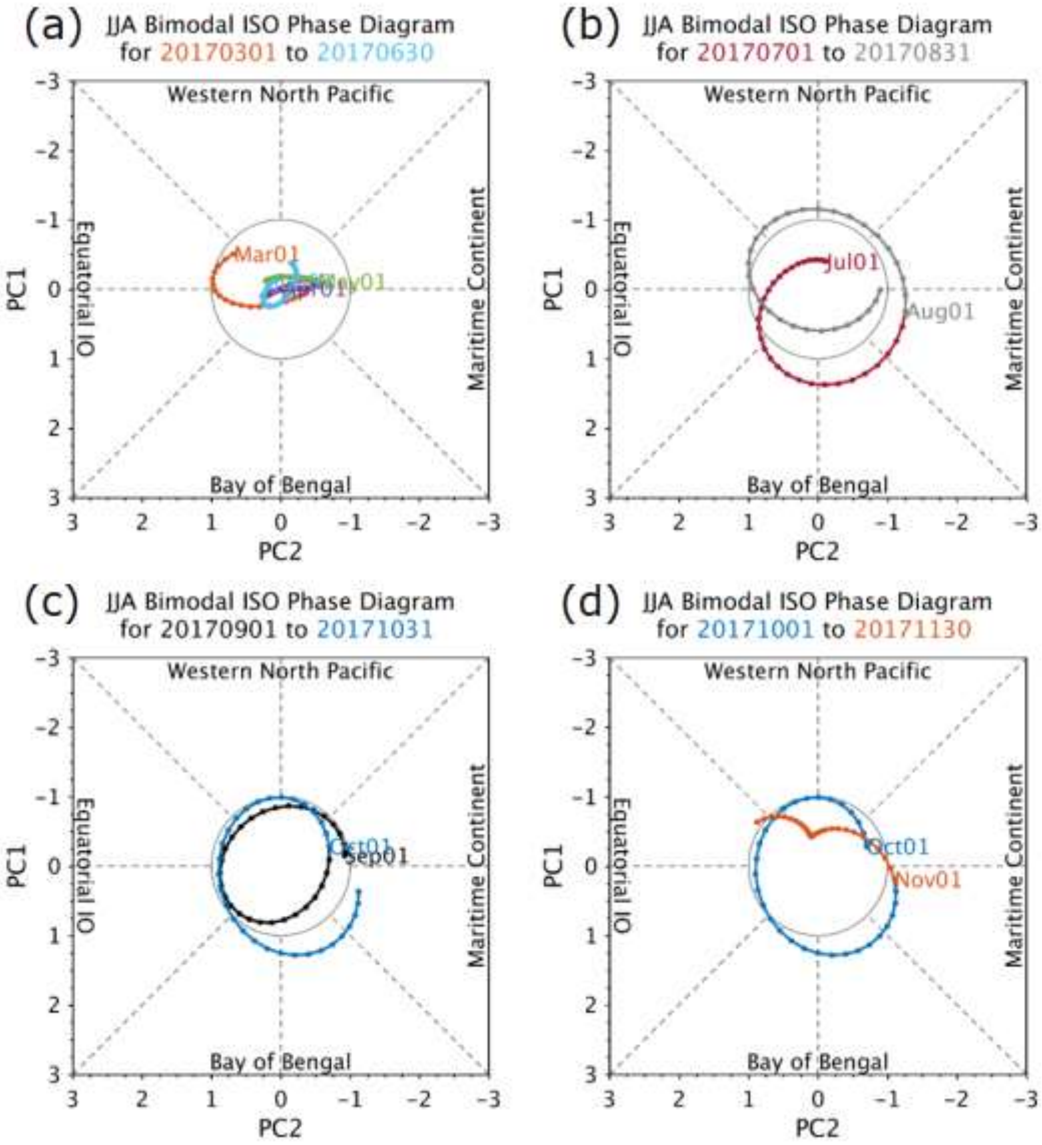

Supplementary Figure 1. The phase diagram for Boreal Summer Intraseasonal Oscillation. Each dot represents one day. The definition is from ref. [3], and the indices are publicly available on website http://iprc.soest.hawaii.edu/users/kazuyosh/Bimodal_ISO.html.

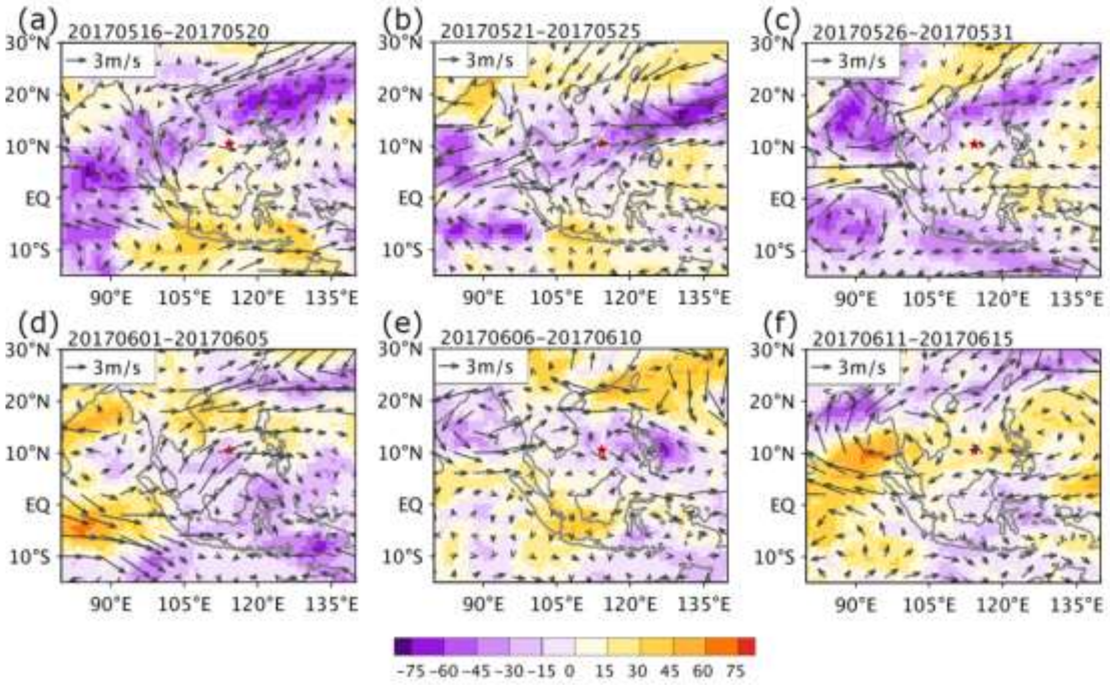

Supplementary Figure 2. The pentad evolution of 8-90 day band-pass filtered outgoing longwave radiation (shading; W m^{-2}) and FNL 850-hPa wind associated with SW1. The red pentagram denotes the position of ADCP.

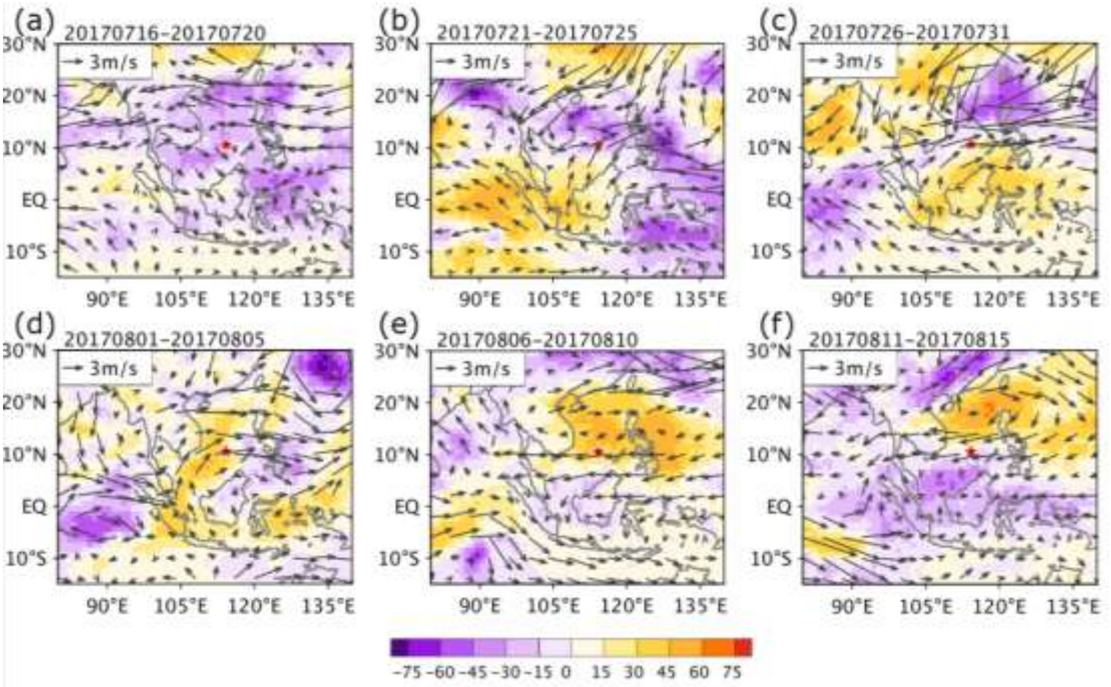

Supplementary Figure 3. Same as Supplementary Figure 2, but for SW2.

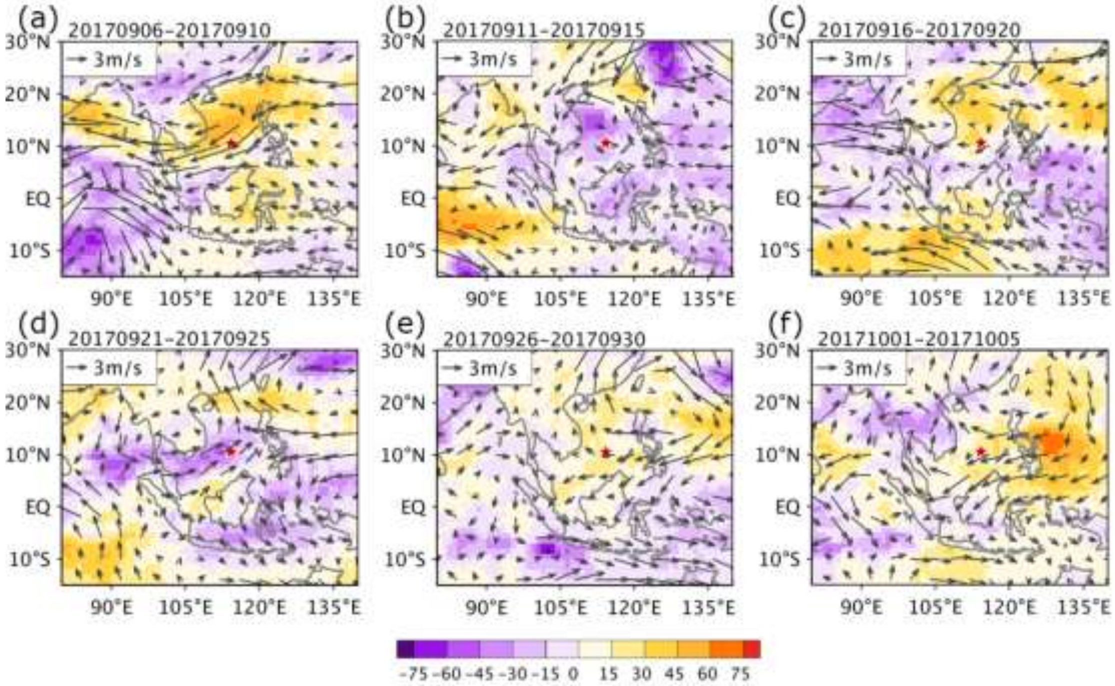

Supplementary Figure 4. Same as Supplementary Figure 2, but for SW3.

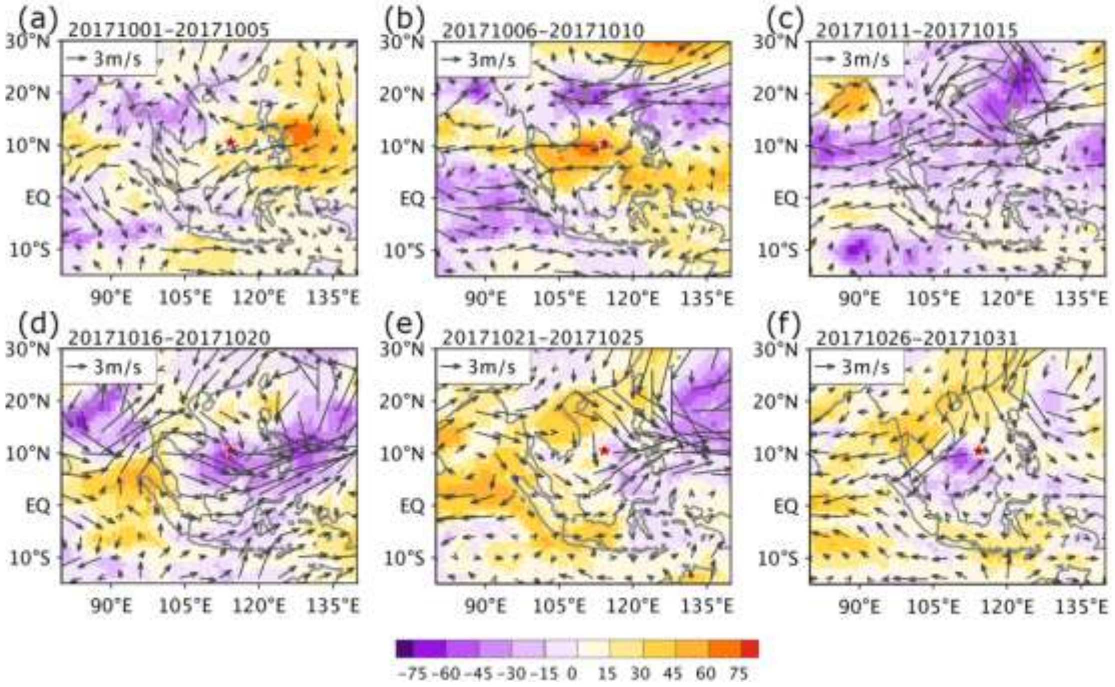

Supplementary Figure 5. Same as Supplementary Figure 2, but for SW4.
